# Distributed model-free formation control of networked fully-actuated autonomous surface vehicles

**DOI:** 10.3389/fnbot.2022.1028656

**Published:** 2022-09-29

**Authors:** Xiaobing Niu, Shengnan Gao, Zhibin Xu, Shiliang Feng

**Affiliations:** ^1^School of Marine Electrical Engineering, Dalian Maritime University, Dalian, China; ^2^China State Shipbuilding Corporation Limited, Beijing, China

**Keywords:** dynamic surface control, adaptive extended state observer, autonomous surface vehicle, model-free control, formation tracking

## Abstract

This paper presents a distributed constant bearing guidance and model-free disturbance rejection control method for formation tracking of autonomous surface vehicles subject to fully unknown kinetic model. First, a distributed constant bearing guidance law is designed at the kinematic level to achieve a consensus task. Then, by using an adaptive extended state observer (AESO) to estimate the total uncertainties and unknown input coefficients, a simplified model-free kinetic controller is designed based on a dynamic surface control (DSC) design. It is proven that the closed-loop system is input-to-state stable The stability of the closed-loop system is established. A salient feature of the proposed method is that a cooperative behavior can be achieved without knowing any priori information. An application to formation control of autonomous surface vehicles is given to show the efficacy of the proposed integrated distributed constant bearing guidance and model-free disturbance rejection control.

## 1. Introduction

In recent years, there has been a surge of interest in distributed cooperative control of autonomous surface vehicles (ASVs). It can be envisioned that multiple ASVs enable vehicles to collaborate with each other to execute difficult missions, contributing to improved efficiency and effectiveness over a single one (Arrichiello et al., 2006; Cui et al., 2010; Peng et al., 2011, 2013, 2020, 2021a,b,c; Wang and Han, 2016; Li et al., 2018; Chen et al., 2020; Guo et al., 2020; Liu et al., 2020a,b, 2022; Zhang et al., 2020; Zhu et al., 2021, 2022; Gu et al., 2022a,b,c,d; Hu et al., 2022a,b; Rout et al., 2022). Recently, distributed control methods have been widely studied (see references, Cao and Ren, 2010; Wang et al., 2010; Zhang et al., 2011, 2012; Cui et al., 2012; Zhang and Lewis, 2012; Hong et al., 2013; Peng et al., 2014; Jiang et al., 2021). In Cao and Ren (2010), a distributed control method is proposed to deal with the formation control problem. In Jiang et al. (2021), a distributed model-free control method is designed using a data-driven fuzzy predictor and extended state observers for ASVs to achieve cooperative target enclosing. A distributed adaptive control method is presented to achieve the cooperative tracking with unknown dynamics in Zhang and Lewis (2012). In Cui et al. (2012), a distributed synchronized tracking control method is designed based on an adaptive neural network for ASVs. In Wang et al. (2010), a distributed control approach is designed to deal with the asymptotic tracking under disturbances generated by the exosystem. A distributed leader-follower control method is proposed using the output regulation theory and internal model principle in Hong et al. (2013). In Peng et al. (2014), a distributed adaptive control method is presented by using the state information of neighboring ASVs only. In Zhang et al. (2011), a distributed control method is presented by using the observer to achieve cooperative tracking. In Zhang et al. (2012), an adaptive distributed control technique is designed based on neural network to deal with the cooperative tracking problems. Its key advantage is that the group objective can be achieved *via* local information exchanges. Consensus-based distributed formation control schemes are presented in Ren (2007), Ren and Sorensen (2008), and Hu (2012). In Ren (2007), a consensus-based distributed control method is proposed to deal with the formation control problem. In Ren and Sorensen (2008), a consensus-based approach is designed to achieve the distributed formation control. In Hu (2012), a distributed consensus-based control method is designed to achieve global asymptotic consensus tracking.

As for autonomous surface vehicle systems, the modeling process is time-consuming and a large number of experiments is required for identifying model parameters. On the other hand, robustness against model uncertainty and ocean disturbances is critical for high-performance control of ASVs (Fossen, 2002; Skjetne et al., 2005; Tee and Ge, 2006; Li et al., 2008; Dai et al., 2012; Chen et al., 2013; How et al., 2013). To deal with this problem, adaptive backstepping and DSC techniques has been widely suggested; see the references (Fossen, 2002; Skjetne et al., 2005; Tee and Ge, 2006; Li et al., 2008; Dai et al., 2012; Chen et al., 2013; How et al., 2013). In Tee and Ge (2006), a stable tracking control method is proposed using backstepping and Lyapunov synthesis for multiple marine vehicles under the unmeasurable states. In Chen et al. (2013), a variable control structure based on backstepping and Lyapunov synthesis is designed for the positioning of marine vessels with the parametric uncertainties and ocean disturbances. In How et al. (2013), an adaptive approximation technique is designed using the backstepping to estimate the uncertainties. In Dai et al. (2012), an adaptive neural networks control method is designed based on the backstepping and Lyapunov synthesis with uncertain environment. In Skjetne et al. (2005), an adaptive recursive control method is designed using the backstepping and Lyapunov synthesis for marine vehicles with the unknown model parameters. Although the adaptive backstepping and DSC are recursive and systematic design methods, it does not offer the freedom to choose the parameter adaptive laws (Krstić et al., 1995). Besides, the identification process depends on the tracking error dynamics, and the transient performance cannot be guaranteed (Cao and Hovakimyan, 2007; Yucelen and Haddad, 2013).

Motivated by the above observations, this article presents a distributed constant bearing guidance and model-free disturbance rejection control method for formation tracking of ASVs subject to fully unknown kinetic model. Specifically, a distributed constant bearing guidance law is designed at the kinematic level to achieve a consensus task. Then, an AESO is constructed for estimating the model uncertainty and unknown ocean disturbances, which can achieve the uncertainty and disturbance estimation. Next, a controller module is developed by using a DSC technique. Simulation results are provided to show the efficacy of the proposed modular design integrated distributed constant bearing guidance and model-free disturbance rejection control method. The main contribution of the proposed control method are stated as follows. Firstly, the proposed design results in the decoupled estimation and control, where the estimation loop is faster than the control loop, yielding the improved transient performance. This contributes to the certainty equivalence control of multi-vehicle systems. Secondly, the security level of ASVs is enhanced by using an AESO to identify the total uncertainties. Finally, the salient feature of the proposed method is that a cooperative behavior can be achieved without knowing any priori information.

The rest of this paper is organized as follows: The problem formulation is presented in Section 2. Section 3 presents the distributed constant bearing guidance and model-free disturbance rejection control method. Section 4 provides simulation results to illustrate the designed model-free disturbance rejection control method for distributed formation tracking. Section 5 concludes this paper.

## 2. Problem formulation

**Figure 1:**
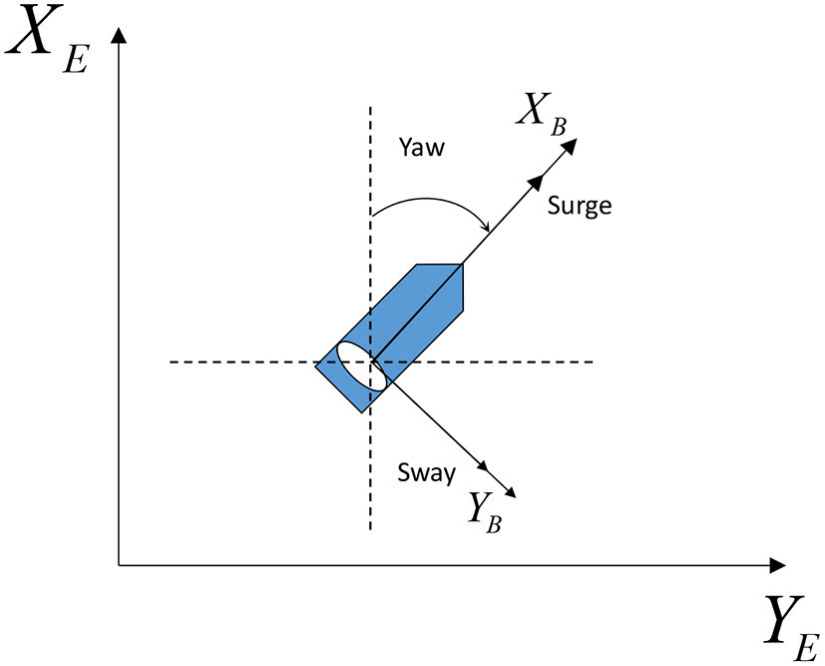
The plane motion diagram of the ASV.

A three degree-of-freedom (DOF) dynamical model for ASVs in a horizontal plane as shown in Figure 1 can be expressed with kinematics (Fossen, 2002; Skjetne et al., 2005).


(1) $$\begin{array}{ll} {\overset{\cdot }{\eta }}_{i} & =R\left(\psi_{i}\right)\nu_{i}, \end{array}$$

(2) $$\begin{array}{ll} {\overset{\cdot }{\nu }}_{i} & =M_{i}^{-1}f_{i}\left(\nu_{i}\right)+M_{i}^{-1}\tau_{i}+M_{i}^{-1}\tau_{wi}\left(t\right), \end{array}$$

where


(3) $$\begin{array}{l} R\left(\psi_{i}\right)=\left[\begin{array}{ccc} \cos\psi_{i} & -\sin\psi_{i} & 0\\ \sin\psi_{i} & \cos\psi_{i} & 0\\ 0 & 0 & 1 \end{array}\right]\;; \end{array}$$

$\eta_{i}={\left[x_{i},y_{i},\psi_{i}\right]}^{T}\in {\mathbb{R}}^{3}$ represents the earth-fixed position and heading; $\nu_{i}={\left[u_{i},v_{i},r_{i}\right]}^{T}\in {\mathbb{R}}^{3}$ includes the body-fixed surge and sway velocities, and the yaw rate; $M_{i}=M_{i}^{T}\in {\mathbb{R}}^{3\times 3},C_{i}\left(\nu_{i}\right)\in {\mathbb{R}}^{3\times 3},D_{i}\left(\nu_{i}\right)\in {\mathbb{R}}^{3\times 3}$ denote the inertia matrix, coriolis/centripetal matrix, and damping matrix, respectively; $\tau_{i}={\left[\tau_{ui},\tau_{vi},\tau_{ri}\right]}^{T}\in {\mathbb{R}}^{3}$ denotes the control input; $\tau_{wi}\left(t\right)={\left[\tau_{wui}\left(t\right),\tau_{wvi}\left(t\right),\tau_{wri}\left(t\right)\right]}^{T}\in {\mathbb{R}}^{3}$ represents the disturbance vector caused by the wind, waves, and ocean currents.

Since the robot dynamics (1) contain unknown dynamics induced by model uncertainty and ocean disturbances, we rewrite the robot kinetics (1) as follows.


(4) $$\begin{array}{l} {\overset{\cdot }{\eta }}_{i}=R\left(\psi_{i}\right)\nu_{i}, \end{array}$$

(5) $$\begin{array}{l} {\overset{\cdot }{\nu }}_{i}=\Lambda_{i}\tau_{i}+s_{i}, \end{array}$$

where


(6) $$\begin{array}{l} s_{i}=M_{i}^{-1}f_{i}\left(\nu_{i}\right)+M_{i}^{-1}\tau_{wi}\left(t\right),\Lambda_{i}=M_{i}^{-1}. \end{array}$$

The *control objective* is to design a cooperative control law τ_*i*_ for ASVs with dynamics (1) to track a reference trajectory η_0_(*t*) such that


(7) $$\begin{array}{l} \underset{t\to \infty }{\lim}||\eta_{i}\left(t\right)-\eta_{0}\left(t\right)||\leq \delta_{i}, \end{array}$$

for some small constant δ_*i*_.

We use the following assumption.

*Assumption 1*: The reference signals η_0_(*t*), ${\overset{\cdot }{\eta }}_{0}\left(t\right)$, and ${\ddot{\eta }}_{0}\left(t\right)$ are bounded.

## 3. Cooperative tracking

In this section, a modular design approach is presented to develop the cooperative formation controllers for ASVs. First, by using the designed AESO to estimate the total uncertainties and fully unknown input coefficients, a simplified model-free dynamic kinematic controller is designed with the aid of a dynamic surface control.

### 3.1. Controller design

*Step 1.* At first, a cooperative tracking error is defined as


(8) $$\begin{array}{l} z_{i1}=R_{i}^{T}\{\sum_{j\in N_{i}}a_{ij}(\eta_{i}-\eta_{j})+a_{i0}(\eta_{i}-\eta_{0})\}, \end{array}$$

where $R_{i}^{T}=R^{T}\left(\psi_{i}\right)$, and *a*_*ij*_ and *a*_*i*0_ are determined by the communication graph, if the *i*th ASV obtains the information of the *j*th, *a*_*ij*_ = 1; otherwise, *a*_*ij*_ = 0. The definition of *a*_*i*0_ is similar to *a*_*ij*_.

*Assumption 2*: The augmented graph contains a spanning tree with the root node being the leader node *n*_0_.

Then, define a global formation tracking error ϵ_*i*_ as


(9) $$\begin{array}{l} \epsilon_{i}=\eta_{i}-\eta_{0}. \end{array}$$

Define L as the Laplacian matrix of the graph and $A_{0}$ as the leader adjacency matrix, which leads to


(10) $$\begin{array}{l} z_{1}=R\left(H⊗I_{3}\right)\epsilon . \end{array}$$

where $H=L+A_{0}$, $z_{1}={\left[z_{i1}^{T},...,z_{iN}^{T}\right]}^{T}$, $\epsilon ={\left[\epsilon_{1}^{T},...,\epsilon_{N}^{T}\right]}^{T}$, and $R=\text{diag}\left\{R_{1}^{T},...,R_{N}^{T}\right\}$. Define *a*_*id*_ = *d*_*i*_+*a*_*i*0_, then, it follows from (1) that the time derivative of *z*_*i*1_ in (8) is obtained


(11) $$\begin{array}{ll} {ż}_{i1}= & -r_{i}Sz_{i1}+a_{id}\nu_{i}-\underset{j\in N_{i}}{\sum }a_{ij}R_{i}^{T}R_{j}\nu_{j}-a_{i0}R_{i}^{T}{\overset{\cdot }{\eta }}_{0}, \end{array}$$

where


(12) $$\begin{array}{l} S=\left[\begin{array}{ccc} 0 & -1 & 0\\ 1 & 0 & 0\\ 0 & 0 & 0 \end{array}\right]. \end{array}$$

A distributed constant-bearing guidance law α_*i*1_ is proposed as follows


(13) $$\begin{array}{l} \alpha_{i1}=\frac{1}{a_{id}}\{-k_{i\eta }\frac{z_{i1}}{\sqrt{z_{i1}^{2}+\Delta^{2}}}+\sum_{j\in N_{i}}a_{ij}R_{i}^{T}R_{j}\nu_{j}+a_{i0}R_{i}^{T}{\dot{\eta }}_{0}\}, \end{array}$$

where Δ is positive constant, and $k_{i\eta }=\text{diag}\left\{k_{i\eta 1},k_{i\eta 2},k_{i\eta 3}\right\}\in {\mathbb{R}}^{3\times 3}$ with *k*_*iη*1_ ∈ ℝ, *k*_*iη*2_ ∈ ℝ, and *k*_*iη*3_ ∈ ℝ being positive constants.

Let us suppose here that α_*i*1_ are unknown, and let it pass through a first-order filter as follows


(14) $$\begin{array}{l} \gamma_{i}{\overset{\cdot }{\nu }}_{id}=\alpha_{i1}-\nu_{id},\nu_{id}\left(0\right)=\alpha_{i1}\left(0\right), \end{array}$$

where γ_*i*_ ∈ ℝ.

Then, the derivative of *q*_*i*_ is obtained as


(15) $$\begin{array}{l} {\overset{\cdot }{q}}_{i}=-\frac{q_{i}}{\gamma_{i}}-{\overset{\cdot }{\alpha }}_{i1}. \end{array}$$

where *q*_*i*_ = α_*i*1_ − ν_*id*_.

Now using (15), we can conclude that


(16) $$\begin{array}{l} q_{i}\left(t\right)=q_{i}\left(0\right)e^{-\frac{t}{\gamma_{i}}}-\int_{0}^{t}e^{-\frac{1}{\gamma_{i}}\left(t-\tau \right)}{\overset{\cdot }{\alpha }}_{i1}\left(\tau \right)d\tau . \end{array}$$

We can obtain that the bound of ||*q*_*i*_(*t*)|| satisfies the following inequality


$$\begin{array}{l} ||q_{i}\left(t\right)||\leq ||q_{i}\left(0\right)||e^{-\frac{t}{\gamma_{i}}}+\alpha_{i1}^{*}\gamma_{i}, \end{array}$$

where $\alpha_{i1}^{*}$ is a positive constant.

*Step 2*: To start with, define the velocity tracking error *z*_*i*2_ as


(17) $$\begin{array}{l} z_{i2}=\nu_{i}-\nu_{id}. \end{array}$$

Take the time derivative of *z*_*i*2_ along (4) is


(18) $$\begin{array}{l} {\overset{\cdot }{z}}_{i2}=\Lambda_{i}\tau_{i}+s_{i}-{\overset{\cdot }{\nu }}_{id}. \end{array}$$

For the robot kinetics (4), an AESO is designed as


(19) $$\begin{array}{l} \{\begin{array}{l} {\overset{\cdot }{\hat{\nu }}}_{i}=\hat{\Lambda }\tau_{i}+{ŝ}_{i}-k_{i\nu }\left({\hat{\nu }}_{i}-\nu_{i}\right),\\ {\overset{\cdot }{ŝ}}_{i}=-k_{is}\left({\hat{\nu }}_{i}-\nu_{i}\right),\\ {\overset{\cdot }{\hat{\sigma }}}_{i}=-\Gamma_{i\sigma }\left({ŝ}_{i}-{\hat{\sigma }}_{i}\right),\\ {\overset{\cdot }{\hat{\Lambda }}}_{i}=-\Gamma_{i\Lambda }\tau \left({ŝ}_{i}-{\hat{\sigma }}_{i}\right), \end{array} \end{array}$$

where $\sigma =s_{i}+\Lambda_{i}\tau_{i}-{\hat{\Lambda }}_{i}\tau_{i}$, $k_{i\nu }=\text{diag}\left\{k_{i\nu 1},k_{i\nu 2},k_{i\nu 3}\right\}\in {\mathbb{R}}^{3\times 3},$ $k_{is}=\text{diag}\left\{k_{is1},k_{is2},k_{is3}\right\}\in {\mathbb{R}}^{3\times 3}$, and *k*_*iν*1_ ∈ ℝ, *k*_*iν*2_ ∈ ℝ, *k*_*iν*3_ ∈ ℝ, *k*_*is*1_ ∈ ℝ, *k*_*is*2_ ∈ ℝ, and *k*_*is*3_ ∈ ℝ are positive constants. ${\hat{\nu }}_{i}$, ŝ_*i*_, ${\hat{\sigma }}_{i}$, and ${\hat{\Lambda }}_{i}$ are the estimates of ν_*i*_, *s*_*i*_, σ_*i*_, and Λ_*i*_, respectively.

*Assumption 3*: For unknown functions *s*_*i*_ and σ_*i*_, there are $s_{i}^{*}\in {ℜ}^{+}$ and $\sigma_{i}^{*}\in {ℜ}^{+}$, such that $||{ṡ}_{i}||\leq s_{i}^{*}$ and $||{\overset{\cdot }{\sigma }}_{i}||\leq \sigma_{i}^{*}$.

Let the parameter estimation be ${\tilde{\Lambda }}_{i}={\hat{\Lambda }}_{i}-\Lambda_{i}$, and the prediction error be ${\tilde{\nu }}_{i}={\hat{\nu }}_{i}-\nu_{i}$. Define ${\tilde{s}}_{i}={ŝ}_{i}-s_{i}$ and ${\tilde{\sigma }}_{i}={\hat{\sigma }}_{i}-\sigma_{i}$. It can be obtained ${\hat{\sigma }}_{i}-{\hat{\Lambda }}_{i}\tau_{i}=-{\tilde{s}}_{i}-{\tilde{\Lambda }}_{i}\tau_{i}+a_{i1}$ with *a*_*i*_ being the reconstruct error. Then, the error dynamics can be expressed as


(20) $$\begin{array}{l} \{\begin{array}{l} {\overset{\cdot }{\tilde{\nu }}}_{i}=-k_{i\nu }{\tilde{\nu }}_{i}+{\tilde{s}}_{i},\\ {\overset{\cdot }{\tilde{s}}}_{i}=-k_{is}{\tilde{\nu }}_{i}-{\dot{s}}_{i},\\ {\overset{\cdot }{\tilde{\sigma }}}_{i}=-\Gamma_{i\sigma }\left({ŝ}_{i}-{\hat{\sigma }}_{i}\right)-{\overset{\cdot }{\sigma }}_{i},\\ {\overset{\cdot }{\tilde{\Lambda }}}_{i}=-\Gamma_{i\Lambda }\tau \left({ŝ}_{i}-{\hat{\sigma }}_{i}\right)-{\overset{\cdot }{\Lambda }}_{i}. \end{array} \end{array}$$

To stabilize *z*_*i*2_, a model-free disturbance rejection control law is proposed as follows


(21) $$\begin{array}{l} \tau_{i}=\frac{-k_{i\tau }z_{i2}+{\overset{\cdot }{\nu }}_{id}-{ŝ}_{i}}{{\hat{\Lambda }}_{i}}, \end{array}$$

where $k_{i\tau }=\text{diag}\left\{k_{i\tau 1},k_{i\tau 2},k_{i\tau 3}\right\}\in {\mathbb{R}}^{3\times 3},$ and $k_{i\tau 1}\in {\mathbb{R}}^{+},$ $k_{i\tau 2}\in {\mathbb{R}}^{+},$ and $k_{i\tau 3}\in {\mathbb{R}}^{+}$.

Substituting (21) into (18) yields


(22) $$\begin{array}{ll} M_{i}{\overset{\cdot }{\hat{z}}}_{i2}= & -k_{i\tau }{\hat{z}}_{i2}-\varrho_{i}{\tilde{\nu }}_{i}, \end{array}$$

where ϱ_*i*_ is a positive constant.

The following lemma presents the stability of AESO error subsystem (20).

*Lemma 1*: Under Assumption 2, the AESO error subsystem (20), viewed as a system with the states being ${\tilde{\nu }}_{i}$, ${\tilde{s}}_{i}$, ${\tilde{\sigma }}_{i}$, and ${\tilde{\Lambda }}_{i}$, the inputs being ṡ_*i*_, ${\overset{\cdot }{\sigma }}_{i}$, and ${\overset{\cdot }{\Lambda }}_{i}$ is ISS.

*Proof* : Construct the Lyapunov function as


(23) $$\begin{array}{l} V_{\sigma i}=\frac{1}{2}\left({\tilde{\sigma }}_{i}^{T}\Gamma_{\sigma i}^{-1}{\tilde{\sigma }}_{i}+{\tilde{\Lambda }}_{i}^{T}\Gamma_{\Lambda i}^{-1}{\tilde{\Lambda }}_{i}\right), \end{array}$$

and the time derivatives of *V*_σ*i*_ is


(24) $$\begin{array}{l} {\overset{\cdot }{V}}_{\sigma i}={\tilde{\sigma }}_{i}\Gamma_{\sigma i}^{-1}\left(-\Gamma_{\sigma i}\left({ŝ}_{i}-{\hat{\sigma }}_{i}\right)-{\overset{\cdot }{\sigma }}_{i}\right)+{\tilde{\Lambda }}_{i}\Gamma_{\Lambda i}^{-1}\left(-\Gamma_{\Lambda i}\left({ŝ}_{i}-{\hat{\sigma }}_{i}\right)\right)\\ \;={\tilde{\sigma }}_{i}\left({ŝ}_{i}-{\hat{\sigma }}_{i}\right)-\Gamma_{\sigma i}^{-1}{\tilde{\sigma }}_{i}{\overset{\cdot }{\sigma }}_{i}+{\hat{\Lambda }}_{i}\tau_{i}\left({ŝ}_{i}-{\hat{\sigma }}_{i}\right)\\ \;\leq -{\tilde{\sigma }}_{i}^{2}-2{\tilde{\sigma }}_{i}{\tilde{\Lambda }}_{i}\tau_{i}-{\tilde{\Lambda }}_{i}^{2}\tau_{i}^{2}-{\tilde{\Lambda }}_{i}\tau_{i}a_{i1}+\Gamma_{\sigma i}^{-1}{\tilde{\sigma }}_{i}\sigma_{i}^{*}\\ \;\leq -||\epsilon_{i}{||}^{2}+||\iota_{i}||||\epsilon_{i}||, \end{array}$$

where $\epsilon_{i}={\tilde{\sigma }}_{i}+{\tilde{\Lambda }}_{i}\tau_{i}$ and $\iota_{i}=\max\left\{a_{i1},\Gamma_{\sigma i}^{-1}\sigma_{i}^{*}\right\}$.

Since


(25) $$\begin{array}{l} ||\epsilon_{i}||\geq ||\iota_{i}||/\theta_{i}, \end{array}$$

renders


(26) $$\begin{array}{l} {\overset{\cdot }{V}}_{\sigma i}\leq -\left(1-\theta_{i}\right)||\epsilon_{i}{||}^{2} \end{array}$$

with θ_*i*_ ∈ (0, 1). Therefore, it can conclude that the error ϵ_*i*_ is bounded.

It follows from (20) that the dynamics of the ${\tilde{\nu }}_{i}$ and ${\tilde{s}}_{i}$ can be rewritten as


(27) $$\begin{array}{l} {\overset{\cdot }{\tilde{\chi }}}_{i}=A_{\chi i}{\tilde{\chi }}_{i}-{\dot{s}}_{\chi i}, \end{array}$$

where ${\tilde{\chi }}_{i}={\left[{\tilde{\nu }}_{i},{\tilde{s}}_{i}\right]}^{T}$, ${ṡ}_{\chi i}={\left[0,{ṡ}_{i}\right]}^{T}$, and


(28) $$\begin{array}{l} A_{\chi i}=\left[\begin{array}{cc} -k_{i\tau } & 1\\ -k_{is} & 0 \end{array}\right], \end{array}$$

with *A*_χ*i*_ being Hurwitz. There exists a unique positive definite matrix *P*_χ*i*_, such that


(29) $$\begin{array}{l} A_{\chi i}^{T}P_{\chi i}+P_{\chi i}^{T}A_{\chi i}=-I. \end{array}$$

Construct the Lyapunov function for system (27) as


(30) $$\begin{array}{l} V_{\chi i}=\frac{1}{2}{\tilde{\chi }}_{i}^{T}P_{\chi i}{\tilde{\chi }}_{i}. \end{array}$$

The dynamics of the *V*_χ*i*_ is


(31) $$\begin{array}{l} {\overset{\cdot }{V}}_{\chi i}={\tilde{\chi }}_{i}^{T}\left(A_{\chi i}^{T}P_{\chi i}+P_{\chi i}^{T}A_{\chi i}\right){\tilde{\chi }}_{i}+{\tilde{\chi }}_{i}^{T}P_{\chi i}\left(-{\dot{s}}_{\chi i}\right)\\ \;\leq -||{\tilde{\chi }}_{i}{||}^{2}+||{\tilde{\chi }}_{i}||||P_{\chi i}||||{\dot{s}}_{\chi i}|| \end{array}$$

Since


(32) $$\begin{array}{l} ||{\tilde{\chi }}_{i}||\geq \left(||P_{\chi i}||||{\dot{s}}_{\chi i}||\right)/a_{i2}, \end{array}$$

renders


(33) $$\begin{array}{l} {\overset{\cdot }{V}}_{\chi i}\leq -\left(1-a_{i2}\right)||{\tilde{\chi }}_{i}{||}^{2} \end{array}$$

with *a*_*i*2_ ∈ (0, 1). It is concluded that the error subsystem (20) is ISS. There exists class KL function β_*i*1_ such that


(34) $$\begin{array}{l} ||\chi_{i}\left(t\right)||\leq \max\left\{\beta_{i1}\left(||\tilde{\chi \left(0\right)}||,t\right),\kappa_{i1}^{s_{i}}\left(||{\dot{s}}_{\chi i}||\right)\right\}, \end{array}$$

with the gain function (Wang et al., 2006) given by


$$\begin{array}{l} \kappa_{i1}^{s_{i}}\left(s\right)=\sqrt{\frac{\lambda_{\max}\left(P_{\chi i}\right)}{\lambda_{\min}\left(P_{\chi i}\right)}}\frac{||P_{\chi i}||s}{a_{i2}}. \end{array}$$

Recalling (11), (17), and (22), the error dynamics is addressed as


(35) $$\begin{array}{l} \{\begin{array}{l} {\dot{z}}_{i1}=-r_{i}Sz_{i1}-k_{i\eta }z_{i1}+a_{id}\left(-{\tilde{\nu }}_{i}+{\hat{z}}_{i2}+q_{i}\right),\\ M_{i}{\overset{\cdot }{\hat{z}}}_{i2}=-k_{i\tau }{\hat{z}}_{i2}-\varrho_{i}{\tilde{\nu }}_{i}, \end{array} \end{array}$$

where *q*_*i*_ = ν_*id*_ − α_*i*1_.

By using the coordinations of *z*_*i*1_ and ẑ_*i*2_, the above subsystem (35) is only perturbed by ${\tilde{\nu }}_{i}$ and *q*_*i*_. Obviously, these two variables will vanish soon as time evolve by choosing the control parameters of predictors and filters.

*Lemma 2*: The error subsystem (35), viewed as a system with the states being *z*_*i*1_ and ẑ_*i*2_ and the inputs being ${\tilde{\nu }}_{i}$ and *q*_*i*_, is ISS.

*Proof* : Construct a Lyapunov function as follows


(36) $$\begin{array}{l} V_{c}=\frac{1}{2}\{z_{i1}^{T}z_{i1}+{\hat{z}}_{i2}^{T}M_{i}{\hat{z}}_{i2}\}. \end{array}$$

Taking the time derivative of *V*_*c*_ along (35), it renders


(37) $$\begin{array}{l} {\overset{\cdot }{V}}_{c}\leq -\lambda_{\min}\left(k_{i\eta }\right)z_{i1}^{T}z_{i1}+a_{id}z_{i1}^{T}\left(-{\tilde{\nu }}_{i}+{\hat{z}}_{i2}+q_{i}\right)\\ \;-\lambda_{\min}\left(k_{i\tau }\right){\hat{z}}_{i2}^{T}{\hat{z}}_{i2}-{\hat{z}}_{i2}^{T}\varrho_{i}{\tilde{\nu }}_{i}. \end{array}$$

Using the inequalities


(38) $$\begin{array}{l} |z_{i1}^{T}{\hat{z}}_{i2}|\leq \frac{1}{2}||z_{i1}{||}^{2}+\frac{1}{2}||{\hat{z}}_{i2}{||}^{2} \end{array}$$

(39) $$\begin{array}{l} |z_{i1}^{T}q_{i}|\leq \frac{1}{2}||z_{i1}{||}^{2}+\frac{1}{2}q_{i}^{*2} \end{array}$$

(40) $$\begin{array}{l} |z_{i1}^{T}{\tilde{\nu }}_{i}|\leq \frac{1}{2}||z_{i1}{||}^{2}+\frac{1}{2}||{\tilde{\nu }}_{i}{||}^{2} \end{array}$$

it follows that


(41) $$\begin{array}{l} {\dot{V}}_{c}\leq -\;(\lambda_{\min}(k_{i\eta })-\frac{3a_{id}}{2})\|z_{i1}{\|}^{2}-\;(\lambda_{\min}(k_{i\tau })\\ \;-\frac{\lambda_{\max}(\varrho_{i})+a_{id}}{2})\|{\hat{z}}_{i2}{\|}^{2}+\frac{\lambda_{\max}(\varrho_{i})+a_{id}}{2}\|{\tilde{\nu }}_{i}{\|}^{2}\\ \;+\frac{a_{id}}{2}\|q_{i}{\|}^{2}. \end{array}$$

By selecting $c_{i}=\min\left\{\lambda_{\min}(k_{i\eta })-\frac{3a_{id}}{2},\lambda_{\min}(k_{i\tau })-\frac{\lambda_{\max}(\varrho_{i})+a_{id}}{2}\right\}>0$ and $Z_{i}=\left[z_{i1}^{T},{ẑ}_{i2}^{T}\right]$, one has


(42) $$\begin{array}{l} {\dot{V}}_{c}\leq -c_{i}\|Z_{i}{\|}^{2}+\frac{\lambda_{\max}(\varrho_{i})+a_{id}}{2}\|{\tilde{\nu }}_{i}{\|}^{2}+\frac{a_{id}}{2}\|q_{i}{\|}^{2}\\ \;\leq -\frac{c_{i}}{2}\|Z_{i}{\|}^{2}-\{\frac{c_{i}}{2}\|Z_{i}{\|}^{2}-\frac{\lambda_{\max}(\varrho_{i})+a_{id}}{2}\|{\tilde{\nu }}_{i}{\|}^{2}\\ \;\;-\frac{a_{id}}{2}\|q_{i}{\|}^{2}\}\;. \end{array}$$

Since


(43) $$\begin{array}{l} \|Z_{i}\|\;\geq \frac{\sqrt{\lambda_{\max}(\varrho_{i})+a_{id}}}{\sqrt{c_{i}}}\|{\tilde{\nu }}_{i}\|+\frac{\sqrt{a_{id}}}{\sqrt{c_{i}}}\|q_{i}\|\;\\ \;\geq \frac{\sqrt{(\lambda_{\max}(\varrho_{i})+a_{id})\|{\tilde{\nu }}_{i}{\|}^{2}+a_{id}\|q_{i}{\|}^{2}}}{\sqrt{c_{i}}}, \end{array}$$

renders


(44) $$\begin{array}{ll} {\overset{\cdot }{V}}_{c} & \leq -\frac{c_{i}}{2}||Z_{i}{||}^{2}. \end{array}$$

There exists class KL function β_*i*2_ such that


(45) $$\begin{array}{l} \|Z_{i}(t)\|\;\leq \max\{\beta_{i2}(||Z_{i}(0)||,t),\kappa_{i1}^{{\tilde{\nu }}_{i}}(\|{\tilde{\nu }}_{i}\|)+\kappa_{i2}^{q_{i}}(\|q_{i}\|)\}, \end{array}$$

where the gain functions are given by


(46) $$\begin{array}{l} \{\begin{array}{l} \kappa_{ic}^{{\tilde{\nu }}_{i}}\left(s\right)=\sqrt{\frac{\lambda_{\max}\left(P_{i2}\right)}{\lambda_{\min}\left(P_{i2}\right)}}\frac{\lambda_{\max}\left(\varrho_{i}\right)+a_{id}}{c_{i}}s\\ \kappa_{ic}^{q_{i}}\left(s\right)=\sqrt{\frac{\lambda_{\max}\left(P_{i2}\right)}{\lambda_{\min}\left(P_{i2}\right)}}\frac{a_{id}}{c_{i}}s \end{array} \end{array}$$

with *P*_*i*2_ = diag{*M*_*i*_, 1}. The proof is completed.

### 3.2. Cascade stability

*Theorem*: Consider the closed-loop network system consisting of the vessels dynamics (1) (2), the AESO (19), the distributed constant-bearing guidance law (13), and the controller (21). If Assumptions 1–3 and *c*_*i*_ > 0 are satisfied, all signals in the closed-loop system are bounded, and the global CFT error ε_*i*_ converges to a neighborhood around zero.

*Proof* : From Lemma 1, we have proved that subsystem (20) with states being $\left({\tilde{\nu }}_{i},{\tilde{s}}_{i}\right)$ and input being ṡ_*i*_ is ISS. From Lemma 2, it can be obtained that subsystem (35) with states being (*z*_*i*1_, ẑ_*i*2_) and inputs being ${\tilde{\nu }}_{i}$ and *q*_*i*_ is ISS. By Krstić et al. (1995), it proves that the cascade system formed by (20) and (35) with states being $\left(z_{i1},{ẑ}_{i2},{\tilde{\nu }}_{i},{\tilde{s}}_{i}\right)$ and the inputs being *q*_*i*_ and ṡ_*i*_ is ISS. Since *q*_*i*_ and ṡ_*i*_ is bounded by $q_{i}^{*}$ and $s_{i}^{*}$, respectively. Then, the error signals $z_{i1},{ẑ}_{i2},{\tilde{\nu }}_{i},$ and ${\tilde{s}}_{i}$ are all bounded. Observing that $\left||z_{i2}||=||-\tilde{\nu_{i}}+{ẑ}_{i2}||\leq ||{\tilde{\nu }}_{i}||+||{ẑ}_{i2}|\right|$, it follows that *z*_*i*2_ is bounded.

Note that as *t* → ∞, β_*i*1_(·) and β_*i*2_(·) → 0, and it follows from (34) and (45) that *z*_*i*1_ is ultimately bounded by


(47) $$\begin{array}{l} \underset{t\to \infty }{\lim}\|z_{i1}\|\;\leq \underset{t\to \infty }{\lim}\|Z_{i}\|,\;\\ \;\leq \kappa_{i1}^{s_{i}}(\|s_{\chi i}\|)+\kappa_{i2}^{q_{i}}(\|q_{i}\|),\\ \;\leq \;\kappa_{i1}^{s_{i}}(s_{i}^{*})+\kappa_{i2}^{q_{i}}(q_{i}^{*}). \end{array}$$

Then, define $\underline{o}(ℋ)$ as the minimal singular value of H, and it follows from Assumption 2 that


(48) $$\begin{array}{l} \|\varepsilon_{i}\|\leq \frac{\left\|z_{1}\right\|}{\underline{o}(ℋ)}. \end{array}$$

From (47) and (48), ε_*i*_ is ultimately bounded as


$$\begin{array}{l} \underset{t\to \infty }{\lim}\|\varepsilon_{i}\|\;\leq \frac{1}{\underline{o}(ℋ)}\sum_{i=1}^{N}\{\kappa_{i1}^{s_{i}}(s_{i}^{*})+\kappa_{i2}^{q_{i}}(q_{i}^{*})\}. \end{array}$$

## 4. An example

**Figure 2:**
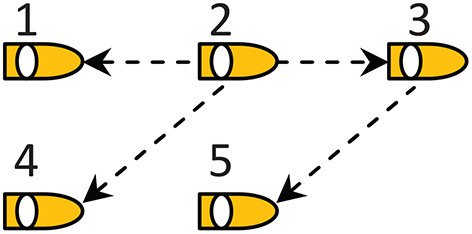
Communication topology.

Consider a networked system consisting of five ASVs, and the communication topology is shown in Figure 2 with the ASV 2 being the leader. The parameters for each model ship are taken from Skjetne et al. (2005). The initial states of five ASVs are set to η_1_ = (0, 0, 0), η_2_ = (0, 12, 0), η_3_ = (0, −12, 0), η_4_ = (0, 24, 0), and η_5_ = (0, −24, 0). In order to better emerge the simulation effect, we add the desired deviations Δ_*ij*_ between the ASVs as follows Δ_12_ = (12, 12, 0), Δ_15_ = (36, 0, 0), Δ_23_ = (8, 8, 0), and Δ_34_ = (8, −8, 0). The control parameters are chosen as *k*_*iη*_ = diag{2, 2, 2}, *k*_*iν*_ = diag{20, 20, 20}, *k*_*is*_ = diag{100, 100, 100}, *k*_*iτ*_ = diag{285, 338, 27.6}, and γ_*i*1_ = 0.02. Define the path variable as ϑ, and the information of path is given in (49)


(49) $$\begin{array}{l} \{\begin{array}{l} {}[0.1\vartheta +20;0;0],\;\vartheta <400,\\ {}[60+60\sin(0.003(\vartheta -400));\\ 60(1-\cos(0.003(\vartheta -400)));\\ 0.003(\vartheta -400)],\\ \vartheta <(400+\pi /0.003);\\ {}[-0.1(\vartheta -400-\pi /0.003)+60;120;\pi ],\\ \vartheta \geq (400+\pi /0.003). \end{array} \end{array}$$

**Figure 3:**
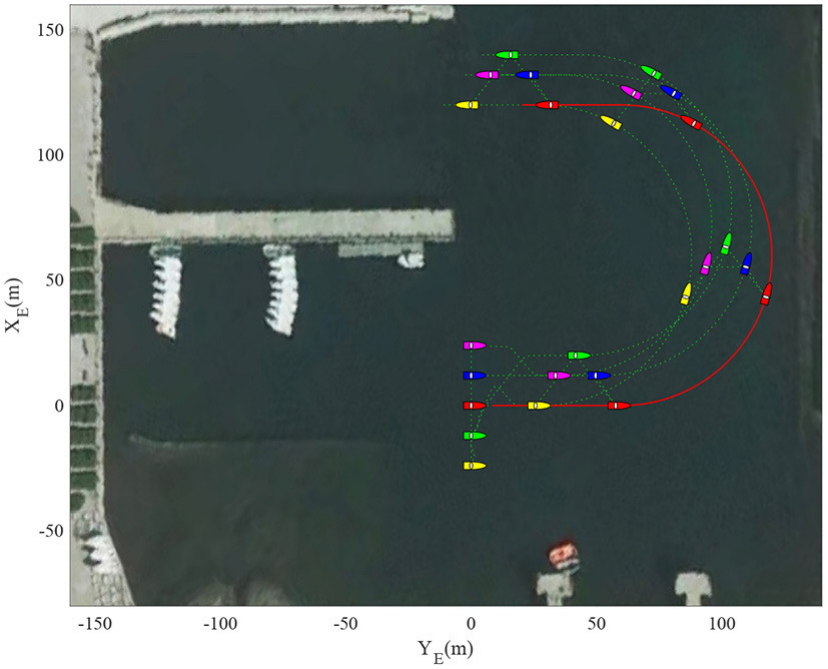
Formation trajectories.

**Figure 4:**
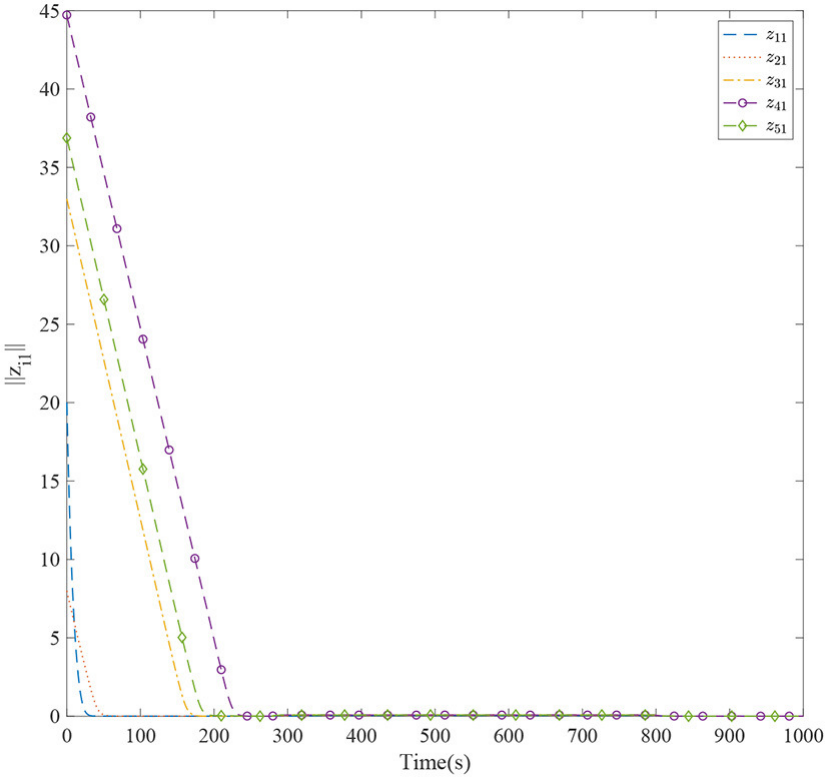
The cooperative tracking errors of five ASVs.

**Figure 5:**
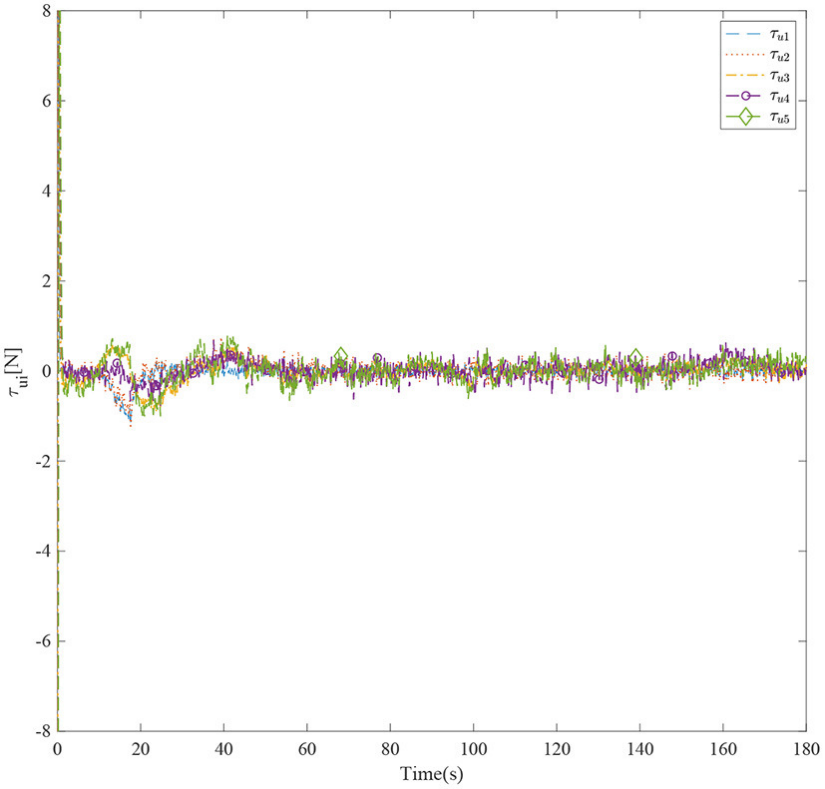
The control inputs τ_*ui*_ of five ASVs.

**Figure 6:**
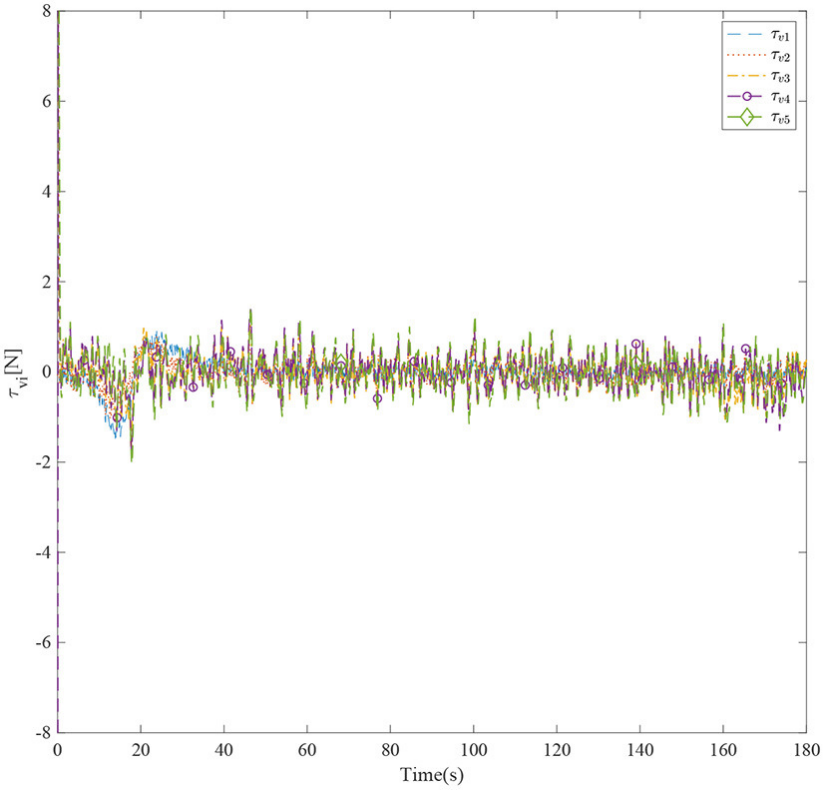
The control inputs τ_*vi*_ of five ASVs.

**Figure 7:**
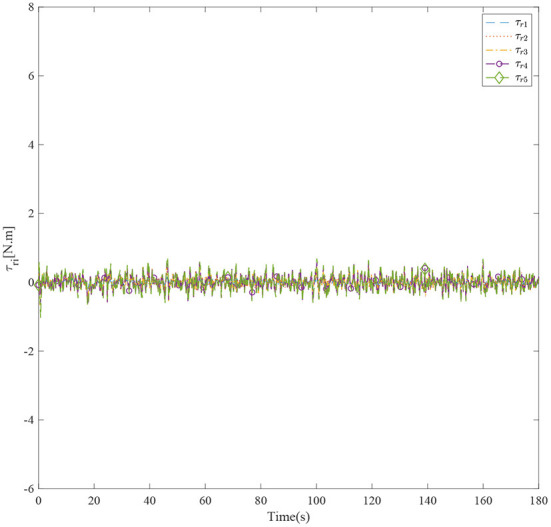
The control inputs τ_*ri*_ of five ASVs.

**Figure 8:**
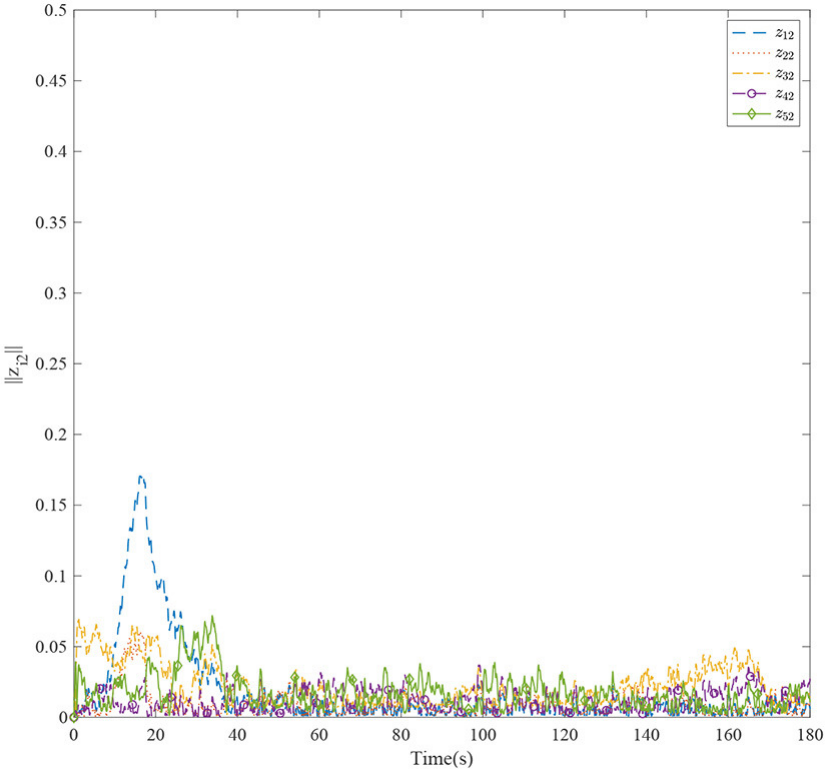
The velocity tracking errors of the five ASVs.

Figure 3 shows the formation trajectories of the five ASVs. It reveals that the a triangle formation can be well established without knowing any priori of the model parameters. Figure 4 shows the cooperative tracking error norms of *z*_*i*1_. It can be seen that the cooperative tracking errors ||*z*_*i*1_|| converge to a neighborhood of the origin. Figures 5–7 show the control inputs in terms of τ_*ui*_, τ_ν*i*_, and τ_*ri*_, respectively. It verifies that the control inputs are all bounded. The velocity tracking error norms of *z*_*i*2_ are shown in Figure 8. It can be seen that the velocity tracking errors ||*z*_*i*2_|| converge to a neighborhood of the origin.

## 5. Conclusions

In this paper, an integrated distributed constant bearing guidance and model-free disturbance rejection control method was presented for cooperative tracking of ASVs subject to fully unknown kinetic model. At the kinematic level, a distributed constant bearing guidance law is designed to achieve a formation task. By using AESO to estimate the total uncertainties and unknown input coefficients, a simplified model-free dynamic kinematic controller is designed with the aid of a dynamic surface control. The stability of the closed-loop cooperative system is proven. The application to formation control of autonomous surface vehicles is given to show the efficacy of the proposed model-free disturbance rejection control method for distributed formation tracking.

## Data availability statement

The original contributions presented in the study are included in the article/supplementary material, further inquiries can be directed to the corresponding author/s.

## Author contributions

Conceptualization, validation, investigation, and writing—review and editing: XN, SG, and ZX. Methodology and resources: XN, SG, and SF. Software and data curation: XN. Formal analysis and writing—original draft preparation: SG. All authors contributed to the article and approved the submitted version.

## Conflict of interest

Author ZX was employed by China State Shipbuilding Corporation Limited. The remaining authors declare that the research was conducted in the absence of any commercial or financial relationships that could be construed as a potential conflict of interest. The reviewer DM declared a shared affiliation with the authors XN, SG, and SF to the handling editor at the time of review.

## Publisher's note

All claims expressed in this article are solely those of the authors and do not necessarily represent those of their affiliated organizations, or those of the publisher, the editors and the reviewers. Any product that may be evaluated in this article, or claim that may be made by its manufacturer, is not guaranteed or endorsed by the publisher.

## References

[B1] Arrichiello F. Chiaverini S. Fossen T. I. (2006). “Formation control of underactuated surface vessels using the Null-Space-Based behavioral control,” in IEEE/RSJ International Conference on Intelligent Robots & *Systems IEEE* (Beijing), 5942–5947. 10.1109/IROS.2006.282477

[B2] Cao C. Y. Hovakimyan N. (2007). Novel *l*_1_ neural network adaptive control architecture with guaranteed transient performance. IEEE Trans. Neural Netw. 18, 1160–1171. 10.1109/TNN.2007.899197 17668668

[B3] Cao Y. Ren W. (2010). Distributed formation control for fractional-order systems: dynamic interaction and absolute/relative damping. Syst. Control Lett. 59, 233–240. 10.1016/j.sysconle.2010.01.008

[B4] Chen L. P. Cui R. Yan W. S. (2020). Adaptive neural network control of underactuated surface vessels with guaranteed transient performance: theory and experimental results. IEEE Trans. Indus. Electron. 67, 4024–4035. 10.1109/TIE.2019.2914631

[B5] Chen M. Ge S. S. How B. V. E. Choo Y. S. (2013). Robust adaptive position mooring control for marine vessels. IEEE Trans. Control Syst. Technol. 21, 395–409. 10.1109/TCST.2012.2183676

[B6] Cui R. Ge S. S. How B. V. E. Choo Y. S. (2010). Leader-follower formation control of underactuated autonomous underwater vehicles. Ocean Eng. 37, 1491–1502. 10.1016/j.oceaneng.2010.07.00632536369

[B7] Cui R. Ren B. Ge S. S. (2012). Synchronised tracking control of multi-agent system with high-order dynamics. IET Control Theory Appl. 6, 603–614. 10.1049/iet-cta.2011.0011

[B8] Dai S. L. Wang C. Luo F. (2012). Identification and learning control of ocean surface ship using neural networks. IEEE Trans. Indus. Inform. 8, 801–810. 10.1109/TII.2012.2205584

[B9] Fossen T. I. (2002). Marine Control System, Guidance, Navigation and Control of Ships, Rigs and Underwater Vehicles. Marine Cyernetics, Trondheim, Norway.

[B10] Gu N. Wang D. Peng Z. Li T. Tong S. (2022a). Model-free containment control of underactuated surface vessels under switching topologies based on guiding vector fields and data-driven neural predictors. IEEE Trans. Cybern. 52, 10843–10854. 10.1109/TCYB.2021.3061588 33822732

[B11] Gu N. Wang D. Peng Z. Wang J. (2022b). Safety-critical containment maneuvering of underactuated autonomous surface vehicles based on neurodynamic optimization with control barrier functions. IEEE Trans. Neural Netw. Learn. Syst. 10.1109/TNNLS.2021.3110014 34520372

[B12] Gu N. Wang D. Peng Z. Wang J. Han Q.-L. (2022c). Disturbance observers and extended state observers for marine vehicles: a survey. Control Eng. Pract. 2022:108258. 10.1016/j.conengprac.2022.105158

[B13] Gu N. Wang D. Peng Z. H. Wang J. Han Q.-L. (2022d). Advances in line-of-sight guidance for path following of autonomous marine vehicles: an overview. IEEE Trans. Syst. Man Cybern. 10.1109/TSMC.2022.3162862

[B14] Guo X. Yan W. S. Cui R. (2020). Modified line-of-sight guidance law with adaptive neural network control of underactuated marine vehicles with state and input constraints. IEEE Trans. Control Syst. Technol. 28, 1902–1914. 10.1109/TCST.2020.2998798

[B15] Hong Y. Wang X. Jiang Z. (2013). Distributed output regulation of leader-follower multi-agent systems. Int. J. Robust Nonlin. Control 23, 48–66. 10.1002/rnc.181436058719

[B16] How B. V. E. Ge S. S. Choo Y. S. (2013). Dynamic load positioning for subsea installation *via* adaptive neural control. IEEE J. Ocean. Eng. 35, 366–375. 10.1109/JOE.2010.2041261

[B17] Hu G. Q. (2012). Robust consensus tracking of a class of second-order multi-agent dynamic systems. Syst. Control Lett. 61, 134–142. 10.1016/j.sysconle.2011.10.004

[B18] Hu X. Wei X. Kao Y. Han J. (2022a). Robust synchronization for under-actuated vessels based on disturbance observer. IEEE Trans. Intell. Transp. Syst. 23, 5470–5479. 10.1109/TITS.2021.3054177

[B19] Hu X. Zhu G. Ma Y. Li Z. Malekian R. Sotelo M. A. (2022b). Event-triggered adaptive fuzzy setpoint regulation of surface vessels with unmeasured velocities under thruster saturation constraints. IEEE Trans. Intell. Transp. Syst. 23, 13463–13472. 10.1109/TITS.2021.3124635

[B20] Jiang Y. Peng Z. Wang D. Yong Y. Han Q.-L. (2021). Cooperative target enclosing of ring-networked under-actuated autonomous surface vehicles based on data-driven fuzzy predictors and extended state observers. IEEE Trans. Fuzzy Syst. 30, 2515–2528. 10.1109/TFUZZ.2021.3087920

[B21] Krstić M. Kanellakopoulos I. Kokotovic P. (1995). Nonlinear and Adaptive Control Design. New York, NY: John Wiley & Sons.

[B22] Li J. Lee P. M. Jun B. H. Lim Y. K. (2008). Point-to-point navigation of underactuated ships. Automatica 44, 3201–3205. 10.1016/j.automatica.2008.08.003

[B23] Li T. Zhao R. Chen C. L. P. Fang L. Liu C. (2018). Finite-time formation control of under-actuated ships using nonlinear sliding mode control. IEEE Trans. Cybern. 48, 3243–3253. 10.1109/TCYB.2018.2794968 29994578

[B24] Liu L. Wang D. Peng Z. Han Q.-L. (2022). Distributed path following of multiple under-actuated autonomous surface vehicles based on data-driven neural predictors *via* integral concurrent learning. IEEE Trans. Neural Netw. Learn. Syst. 32, 5334–5344. 10.1109/TNNLS.2021.3100147 34357868

[B25] Liu L. Wang D. Peng Z. Li T. Chen C. L. P. (2020a). Cooperative path following of ring-networked under-actuated autonomous surface vehicles: algorithms and experiment results. IEEE Trans. Cybern. 50, 1519–1529. 10.1109/TCYB.2018.2883335 30530352

[B26] Liu L. Zhang W. Wang D. Peng Z. (2020b). Event-triggered extended state observers design for dynamic positioning vessels subject to unknown sea loads. Ocean Eng. 2020:107242. 10.1016/j.oceaneng.2020.107242

[B27] Peng Z. Liu L. Wang J. (2021a). Output-feedback flocking control of multiple autonomous surface vehicles based on data-driven adaptive extended state observers. IEEE Trans. Cybern. 51, 4611–4622. 10.1109/TCYB.2020.3009992 32816683

[B28] Peng Z. Wang D. Chen Z. Hu X. Lan W. (2013). Adaptive dynamic surface control for formations of autonomous surface vehicles with uncertain dynamics. IEEE Trans. Control Syst. Technol. 21, 513–520. 10.1109/TCST.2011.2181513

[B29] Peng Z. Wang D. Hu X. (2011). Robust adaptive formation control of underactuated autonomous surface vehicles with uncertain dynamics. IET Control Theory Appl. 5, 1378–1387. 10.1049/iet-cta.2010.0429

[B30] Peng Z. Wang D. Li T. Han M. (2020). Output feedback cooperative formation maneuvering of autonomous surface vehicles with connectivity preservation and collision avoidance. IEEE Trans. Cybern. 50, 2527–2535. 10.1109/TCYB.2019.2914717 31180878

[B31] Peng Z. Wang D. Wang J. (2021b). Data-driven adaptive disturbance observers for model-free trajectory tracking control of maritime autonomous surface ships. IEEE Trans. Neural Netw. Learn. Syst. 32, 5584–5593. 10.1109/TNNLS.2021.3093330 34255635

[B32] Peng Z. Wang D. Zhang H. Sun G. (2014). Distributed neural network control for adaptive synchronization of uncertain dynamical multiagent systems. IEEE Trans. Neural Netw. Learn. Syst. 25, 1508–1519. 10.1109/TNNLS.2013.2293499 25050948

[B33] Peng Z. Wang J. Wang D. Han Q.-L. (2021c). Automatic leader-follower persistent formation control for autonomous surface vehicles. IEEE Trans. Indus. Informatics 17, 732–745. 10.1109/TII.2020.3004343

[B34] Ren W. (2007). Consensus strategies for cooperative control of vehicle formations. IET Control Theory Appl. 1, 505–512. 10.1049/iet-cta:20050401

[B35] Ren W. Sorensen N. (2008). Distributed coordination architecture for multi-robot formation control. Robot. Auton. Syst. 56, 324–333. 10.1016/j.robot.2007.08.005

[B36] Rout R. Cui R. Yan W. S. (2022). Sideslip-compensated guidance-based adaptive neural control of marine surface vessels. IEEE Trans. Cybern. 52, 2860–2871. 10.1109/TCYB.2020.3023162 33055044

[B37] Skjetne R. Fossen T. I. Kokotovic P. V. (2005). Adaptive maneuvering, with experiments, for a model ship in a marine control laboratory. Automatica 41, 289–298. 10.1016/j.automatica.2004.10.006

[B38] Tee K. P. Ge S. S. (2006). Control of fully actuated ocean surface vessels using a class of feedforward approximators. IEEE Trans. Control Syst. Technol. 14, 750–756. 10.1109/TCST.2006.872507

[B39] Wang C. Hill D. J. Ge S. S. Chen G. R. (2006). An ISS-modular approach for adaptive neural control of pure-feedback systems. Automatica 42, 723–731. 10.1016/j.automatica.2006.01.004

[B40] Wang X. Hong Y. Huang J. Jiang Z. P. (2010). A distributed control approach to a robust output regulation problem for multi-agent linear systems. IEEE Trans. Autom. Control 55, 2891–2895. 10.1109/TAC.2010.2076250

[B41] Wang Y.-L. Han Q.-L. (2016). Network-based fault detection filter and controller coordinated design for unmanned surface vehicles in network environments. IEEE Trans. Indus. Inform. 12, 1753–1765. 10.1109/TII.2016.2526648

[B42] Yucelen T. Haddad W. M. (2013). Low-frequency learning and fast adaptation in model reference adaptive control. IEEE Trans. Autom. Control 58, 1080–1085. 10.1109/TAC.2012.2218667

[B43] Zhang H. Lewis F. L. Das A. (2011). Optimal design for synchronization of cooperative systems: state feedback, observer and output feedback. IEEE Trans. Autom. Control 56, 1948–1952. 10.1109/TAC.2011.2139510

[B44] Zhang H. Lewis F. L. Qu Z. H. (2012). Lyapunov, adaptive, and optimal design techniques for cooperative systems on directed communication graphs. IEEE Trans. Indus. Electron. 59, 3026–3041. 10.1109/TIE.2011.2160140

[B45] Zhang H. W. Lewis F. L. (2012). Adaptive cooperative tracking control of higher-order nonlinear systems with unknown dynamics. Automatica 48, 1432–1439. 10.1016/j.automatica.2012.05.008

[B46] Zhang X.-M. Han Q.-L. Ge X. Ding D. Ding L. Yue D.. (2020). Networked control systems: a survey of trends and techniques. IEEE/CAA J. Autom. Sin. 7, 1–17. 10.1109/JAS.2019.1911651

[B47] Zhu G. Ma Y. Li Z. Malekian R. Sotelo M. (2021). Adaptive neural output feedback control for MSVs with predefined performance. IEEE Trans. Vehicul. Technol. 70, 2994–3006. 10.1109/TVT.2021.3063687

[B48] Zhu G. Ma Y. Li Z. Malekian R. Sotelo M. (2022). Event-triggered adaptive neural fault-tolerant control of underactuated MSVs with input saturation. IEEE Trans. Intell. Transp. Syst. 23, 7045–7057. 10.1109/TITS.2021.3066461

